# Dental caries, oral hygiene and salivary characteristics in children with chronic kidney disease: a case–control study

**DOI:** 10.1007/s00467-025-06730-4

**Published:** 2025-03-19

**Authors:** Nur Kodaman Dokumacıgil, Berkant Sezer, Şehkar Oktay, Harika Alpay, Betül Kargül

**Affiliations:** 1 Department of Pediatric Dentistry, Faculty of Dentistry, Institute of Health Science, Marmara University, Istanbul, Türkiye; 2 Department of Pediatric Dentistry, School of Dentistry, Marmara University, Istanbul, Türkiye; 3 Department of Pediatric Dentistry, School of Dentistry, Çanakkale Onsekiz Mart University, Çanakkale, Türkiye; 4 Department of Biochemistry, School of Dentistry, Marmara University, Istanbul, Türkiye; 5 Division of Pediatric Nephrology, Department of Pediatrics, School of Medicine, Marmara University, Istanbul, Türkiye; 6 School of Dentistry, Queen Mary University of London, London, UK

**Keywords:** Biomarkers, Children, Chronic kidney disease, Dental caries, Oral hygiene, Saliva

## Abstract

**Background:**

The aim of this study was to compare the oral health findings and salivary parameters of children with different stages of chronic kidney disease (CKD) with those of healthy peers.

**Methods:**

Intraoral examinations were performed on 43 children aged 8–17 years with CKD and 40 healthy controls from the same pediatric nephrology clinic. Oral health was assessed using the DMFT/dft indices (decayed-missing-filled-teeth), debris index (DI), calculus index (CI), and simplified oral hygiene index (OHI-S). Saliva samples from the children were analyzed for salivary flow rate (SFR), pH, buffering capacity (BC), total oxidant status (TOS), total antioxidant capacity (TAOC), urea, creatinine (Cr), calcium (Ca), potassium (K), phosphorus (P), and salivary α-amylase (SAA). Spearman’s *rho* coefficient was used to examine the relationship between salivary and serum biomarkers levels and oral health findings.

**Results:**

While the DMFT/dft scores were lower in children with CKD (*p* = 0.001), DI, CI, and OHI-S scores were higher in healthy peers (*p* < 0.001). Children with CKD had lower SFR, Ca, and TAOC levels, and higher BC, pH, urea, Cr, K, P, TOS, and SAA levels (*p* < 0.001) compared to healthy controls. Later stages of CKD was associated with the lower dft (${r}_{s}$= − 0.35; *p* = 0.022).

**Conclusions:**

Children with CKD exhibit fewer caries and poorer oral hygiene compared to their healthy peers, and their saliva characteristics differ significantly from those of the healthy group. Disease-related changes in serum and salivary characteristics affect the oral health of children with CKD, necessitating collaboration between pediatric nephrologists and dentists.

**Trial registration:**

ClinicalTrials.gov (NCT06578832).

**Graphical abstract:**

**Figure:**
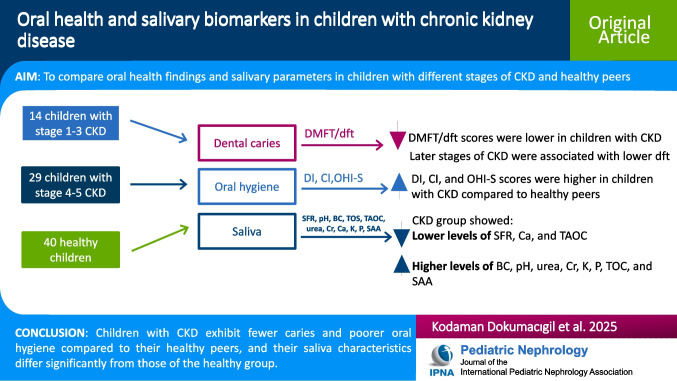
A higher-resolution version of the Graphical abstract is available as Supplementary information

**Supplementary Information:**

The online version contains supplementary material available at 10.1007/s00467-025-06730-4.

## Introduction

Chronic kidney disease (CKD) is a multisymptomatic syndrome characterized by progressive and irreversible damage to or loss of functional nephrons, leading to a sustained decrease in glomerular filtration rate (GFR) for over 3 months [1]. CKD is associated with multi-organ involvement and the accumulation of metabolic waste products, which disrupt the kidney’s endocrine functions. These disruptions include impaired electrolyte and water regulation, acid–base imbalance, elevated blood urea and creatinine levels, and a reduced GFR [2].

The natural course of CKD, along with the side effects of medications used in its treatment, can result in oral complications such as craniofacial growth disturbances, developmental defects of enamel (DDE), gingivitis, gingival overgrowth, xerostomia, halitosis, stomatitis, and dysgeusia [3–6].

Saliva, a biological fluid regulated by both the sympathetic and parasympathetic nervous systems, plays crucial roles in taste perception, chewing, and swallowing, as well as in protecting and cleansing the oral mucosa, facilitating tissue healing, and promoting enamel remineralization [7, 8]. It contains a diverse array of biological components, including minerals, hormones, enzymes, immunoglobulins, cytokines, and antimicrobial peptides, making its analysis and metabolic profiling a valuable diagnostic tool [9]. Metabolic byproducts from bodily processes enter saliva via blood ultrafiltration, often altering both its flow rate and composition [10, 11]. Due to its non-invasive and painless collection process, saliva is particularly favored as a diagnostic material for use in children [12].

Children with CKD typically exhibit a reduced salivary flow rate, with their saliva containing higher concentrations of urea, creatinine (Cr), phosphorus (P), and potassium (K), along with lower calcium (Ca) levels, and increased pH and buffering capacity compared to healthy individuals. These salivary changes can disrupt the balance of the oral environment, potentially leading to oral dysfunction, calculus formation, and an increased risk of dental caries and periodontal disease prevalence [13].

Impaired kidney function leads to increased oxidative stress and/or excessive production of pro-inflammatory cytokines, resulting in the accumulation of uremic toxins [14]. Although the role of oxidative stress in salivary gland dysfunction among children with CKD is well-documented [15, 16], the exact cause of salivary gland hypofunction remains unclear. The literature contains a limited number of studies examining the relationship between oral health and salivary pathogenesis in children with CKD [17]. Additionally, understanding the effects of CKD on salivary flow rate and ionic composition can aid dental professionals in planning treatment, enabling more accurate diagnoses, tailored medication prescriptions, and specific oral health guidance for these patients [18]. Therefore, the main objective of our study was to comparatively evaluate the effect of various biomarkers in saliva on the oral health status of children with CKD at different stages of the disease, compared with healthy children. Another aim was to investigate the correlation between the ionic composition of saliva and plasma in children with CKD, in light of the growing interest in using saliva as a diagnostic material in recent years [14, 19]. The primary null hypothesis of the study was that there would be no significant difference in oral health and salivary parameters between children with CKD and healthy controls. The secondary null hypothesis was that there would be no significant correlation between serum and salivary biomarker levels.

## Methods

### Study design

This observational cohort study is a single-center, controlled study that evaluated the oral health and salivary biomarker levels of children with CKD at different stages. This study was conducted at the Pediatric Nephrology Clinic, Department of Pediatrics, Istanbul Pendik Education and Research Hospital, School of Medicine, Marmara University. The study was conducted in accordance with the Declaration of Helsinki, and it was approved by the Marmara University School of Medicine Clinical Research Ethics Committee (with protocol number: 09.2022.961) and was registered at ClinicalTrials.gov as NCT06578832. The study details and planned examinations were communicated to the participants in a manner appropriate to their age, understanding, and decision-making abilities. Comprehensive information was also provided to their parents and/or caregivers, and their written informed consent was obtained after explaining the study to both the patients and their parents and/or caregivers.

### Sample size calculation

The minimum sample size was calculated based on comparisons of saliva between two groups through Student’s *t*-tests. According to G*Power software (v. 3.1.9.7, Universitat Kiel, Kiel, Germany), with 95% confidence (1-α), 90% power (1-β), and *d* = 0.8 effect size, the number of samples to be taken in each group was calculated as 34, and the total number of samples was calculated as 68.

### Study population

This study enrolled 40 children aged 8–17 years with CKD who were followed up for at least 1 year by the Pediatric Nephrology Clinic, Department of Pediatrics, Istanbul Pendik Education and Research Hospital, School of Medicine, Marmara University, between July 2022 and December 2023. CKD was diagnosed based on the 2012 Kidney Disease Improving Global Outcomes (KDIGO) diagnostic criteria [2]. CKD is classified into five stages based on GFR: stage 1, where GFR is within the normal range (≥ 90 mL/min per 1.73 m^2^) but may show renal parenchymal damage; stage 2, where GFR is slightly reduced; and when GFR falls below 60 mL/min per 1.73 m^2^, moderate and severe renal disease and renal failure may develop (stages 3 to 5). In stage 5 (≥ 15 mL/min/1.73 m^2^), kidney replacement therapy (hemodialysis, peritoneal dialysis, kidney transplant) may be required [20]. To determine the stages of CKD for the classification of study groups, the estimated glomerular filtration rate (eGFR) was calculated using the original Schwartz formula, expressed as follows: eGFR (mL/min/1.73 m^2^) = K × (height in cm / sCr in mg/dL), where *K* is 0.45 for infants (< 1 year), 0.55 for children and adolescent females, and 0.70 for adolescent males (≥ 13 years) [21]. Based on this classification, patients were divided into two groups: stages 1–3 and stages 4–5, reflecting the asymptomatic nature of stages 1–3 with relatively preserved kidney function and the symptomatic presentation and significant renal dysfunction observed in stages 4–5 [4, 22]. Forty-three children who attended the relevant clinic for routine follow-up and were diagnosed with CKD according to KDIGO diagnostic criteria were included in the study.

The control group comprised children who visited the nephrology clinic for routine health check-ups or due to temporary symptoms, such as urinary tract infections or other non-chronic conditions. Following detailed examinations and laboratory tests, a specialist pediatric nephrologist confirmed that these children did not have CKD, any other chronic renal diseases, or systemic illnesses. The healthy children had normal serum Cr levels and urine analyses, no history of urinary tract disease, hypertension, diabetes, or other cardiovascular diseases and were not taking any medications [18]. Children with a history of renal transplantation, any comorbid diseases or conditions, radiation therapy for head and neck cancer, or periodontal treatment within the previous 6 months were excluded from the study [18, 23].

### Clinical data

The study collected demographic and health-related characteristics, including age, sex, CKD stage, and recent blood test results for serum urea (mg/dl), Cr (mg/dl), Ca (mg/dl), and K (mg/dl). Additionally, oral hygiene habits, such as the frequency of dental visits, tooth brushing, and flossing, were recorded. The patient’s oral examination was performed during their routine CKD follow-up appointment. Blood test results, obtained as part of standard clinical care and unrelated to the study, were analyzed to assess the disease’s current state and treatment effectiveness.

### Intraoral examination

All children in both the CKD and healthy groups were examined by the same pediatric dentist (N.K.D). Dental examinations for both groups included assessments of caries (d/D), missing (M), and filled (f/F) teeth (t/T) for primary (dft) and permanent (DMFT) dentition. These evaluations were conducted using standardized measurement criteria with a dental pen light (Dentmate Lumindex 3, China), plane mirror (#5), and dental probe (PCP 11, Hu-Friedy, Chicago, IL, USA), in accordance with the World Health Organization 2013 criteria [24]. Due to the uncertainty surrounding the exact reasons for the loss of primary teeth, the M (missing) component of the DMFT index was not applied in the dft index [24].

The simplified oral hygiene index (OHI-S) was used to evaluate oral hygiene, plaque, and calculus. This index consists of two subcomponents: the debris index (DI) and the calculus index (CI). For each participant, debris and calculus scores were recorded on the buccal/vestibular surfaces of the maxillary right central incisor (#11), maxillary right (#16) and left (#26) first molars, mandibular left central incisor (#31), and the lingual/palatal surfaces of the mandibular left (#36) and right (#46) first molars. These scores were averaged by dividing the total for each subcomponent by the number of examined surfaces, yielding individual DI and CI scores. The overall OHI-S score was calculated by summing the DI and CI scores, with higher scores indicating poorer oral hygiene [24].

### Assessment of salivary biomarkers, flow rate, pH, and buffering capacity

Unstimulated saliva was collected using the spitting method between 8 a.m. and 10 a.m. Patients were instructed to brush their teeth in the morning and then come fasting, without eating, drinking, or chewing gum. Saliva samples were collected in a quiet environment to avoid stress. Patients spat saliva into plastic cups every 30 s, while in an upright position, with their heads slightly tilted forward and their lips closed [16]. To collect stimulated saliva samples, participants were asked to chew odorless, tasteless, 5 mg paraffin chewing tablets to activate salivary secretion. After chewing for 30 s, participants were asked to spit the collected saliva into a separate container. They then continued to chew the paraffin for another 5 min, spitting the collected saliva into a sterile Falcon tube every 15–20 s [18]. The saliva flow rate was measured with an electronic scale (1 mg/min = 1 mL/min). The pH and buffering capacity of unstimulated saliva were determined using pH strips and colored indicator paper from the Saliva Check Buffer kit (GC, Tokyo, Japan). Unstimulated whole saliva samples from all subjects were collected and analyzed for urea (mg/dl) (Cat no: E-BC-K183-S), Cr (mg/dl) (Cat no: E-EL-0058), Ca (mg/dl) (Cat no: E-BC-K103-M), K (mg/dl) (Cat no: E-BC-K279-M), P (mg/dl) (Cat no: E-BC-K245-M), total oxidant status (TOS) (μmol H_2_O_2_/L) (Cat no: E-BC-K802-M), and total antioxidant capacity (TAOC) (mmol Trolox/L) (Cat no: E-BC-K801-M) using the kit method according to the manufacturer’s instructions (Elabscience Biotechnology Co., Ltd., Wuhan, China). Additionally, salivary alpha-amylase (SAA) activity (U/ml) was determined using an SAA assay kit (Cat no: 1–1902, 96, Salimetrics LLC, USA) following the protocol provided by the manufacturer.

### Statistical analysis

The descriptives were presented as mean ± standard deviation (SD) and median (IQR) for the continuous variables, and frequency (%) was reported for the categorical variables. The normal distribution and homogeneity of variances assumptions were examined using Shapiro–Wilk test and Levene test, respectively. The group differences were analyzed with Mann–Whitney *U* test, independent samples *T*-test, Welch’s *T*-test, chi-square test, and Yates continuity correction. Spearman’s rank correlation analysis was conducted to investigate the relationships between CKD patients. The statistical significance was set at 0.05 level. The analysis was carried out in the open-source program JASP software (Version 0.16.3.0; University of Amsterdam, Holland).

## Results

The mean ages, age and sex characteristics, and oral hygiene habits of children with CKD and healthy children are summarized in Table 1. This study included 43 children diagnosed with CKD (51.8%) and 40 healthy children (48.2%). The CKD group comprised 22 boys (51.2%) and 21 girls (48.8%), while the control group included 23 boys (57.5%) and 17 girls (42.5%). The mean age was 12.00 ± 2.49 years in children with CKD and 11.05 ± 2.00 years in healthy children (*p* > 0.05). No significant differences in age or sex were observed between the two groups. However, oral hygiene habits, including the frequency of tooth brushing, dental flossing, and regular dental visits, were significantly poorer in children with CKD compared to healthy children (*p* < 0.001).

**Table 1:** Age, sex, and oral hygiene habits of children with chronic kidney disease (CKD) and healthy children

Variables		CKD (*n* = 43)	Healthy (*n* = 40)	*p*-value
Age (mean ± SD)		12.00 ± 2.49	11.05 ± 2.00	0.059^a^
Median (IQR)		12.0 (10.0–14.0)	11.0 (10.0–12.0)	
Sex, *n* (%)	Male	22 (51.2%)	23 (57.5%)	0.563^b^
	Female	21 (48.8%)	17 (42.5%)	
Oral hygiene habits, *n* (%)				
Toothbrushing	Never	20 (46.5%)	4 (10.0%)	** < 0.001**^b*^
	Once a day	17 (39.5%)	13 (32.5%)	
	Twice a day or more	6 (14.0%)	23 (57.5%)	
Dental floss using	Yes No	1 (2.3%) 42 (97.7%)	18 (45.0%) 32 (55.0%)	** < 0.001**^c*^
Regular dental visits	No	25 (58.1%)	3 (7.5%)	** < 0.001**^b*^
	Yes	18 (41.9%)	37 (92.5%)	

^a^Mann–Whitney *U* test. ^b^Chi-square test. ^c^Yates continuity correction. Two-tailed *p*-value *significant at 0.05 level

The DMFT (*p* < 0.001) and dft (*p* < 0.05) index scores in CKD patients were significantly lower than in healthy children. The poor oral hygiene indicators, including DI, CI, and OHI-S scores, were significantly higher in children with CKD compared to their healthy peers (*p* < 0.001). The unstimulated whole salivary flow rate and stimulated whole salivary flow rate were significantly lower in children with CKD than in healthy children (*p* < 0.001). The salivary buffer capacity and pH levels were significantly higher in children with CKD compared to healthy peers (*p* < 0.001). Saliva urea, Cr, K, P, TOS, and SAA levels were higher, while Ca and TAOC levels were lower in children with CKD compared to the healthy group (*p* < 0.05) (Table 2).

**Table 2:** Dental caries presence**,** DMFT/dft and debris, calculus, simplified oral hygiene index scores, and salivary characteristics in children with chronic kidney disease and healthy children

Variables	CKD (*n* = 43)		Healthy (*n* = 40)		*p*-value
*Oral health parameters*					
*Dental caries presence, n (%)*					
Yes	14 (32.5%)		34 (85%)		** < 0.001**^c*^
No	29 (67.5%)		6 (15%)		
	***Mean* ± *SD***	***Median (IQR)***	***Mean* ± *SD***	***Median (IQR)***	
DMFT	0.84 ± 1.53	0 (0–1)	4.9 ± 3.87	4 (2.5–6)	** < 0.001**^a*^
dft	1.33 ± 2.59	0 (0–1)	2.13 ± 2.46	1 (0–4)	**0.021**^a*^
DI	1.64 ± 0.61	1.7 (1.2–2)	0.38 ± 0.32	0.3 (0.1–0.7)	** < 0.001**^a*^
CI	1.04 ± 0.7	1 (0.5–1.5)	0.07 ± 0.12	0 (0–0.2)	** < 0.001**^a*^
OHI-S	2.7 ± 1.24	2.8 (1.7–3.7)	0.46 ± 0.41	0.3 (0.1–0.7)	** < 0.001**^a*^
*Salivary characteristics*					
*UWS (mL/min)*	0.31 ± 0.16	0.3 (0.2–0.4)	0.59 ± 0.15	0.6 (0.5–0.7)	** < 0.001**^a*^
*SWS (mL/min)*	0.54 ± 0.2	0.5 (0.4–0.7)	0.94 ± 0.26	0.9 (0.7–1.1)	** < 0.001**^a*^
*SBC*	10.14 ± 2	10 (10–12)	8.1 ± 2.35	7.5 (7–10.5)	** < 0.001**^a*^
*SpH*	7.42 ± 0.35	7.4 (7.2–7.6)	7.05 ± 0.39	7 (6.8–7.3)	** < 0.001**^a*^
*Urea (mg/dl)*	12.3 ± 7.35	12 (5–17.6)	4.95 ± 2.74	4.8 (2.6–7.5)	** < 0.001**^a*^
*Cr (mg/dl)*	7.70 ± 1.76	7.5 (6.2–8.5)	6.75 ± 1.54	6.3 (5.6–8.2)	**0.023**^a*^
*Ca (mg/dl)*	3.98 ± 2.24	3.3 (2.5–5.7)	5.52 ± 2.04	5.3 (4.1–6.9)	** < 0.001**^a*^
*K (mg/dl)*	9.41 ± 3.87	7.7 (7.2–10.5)	5.85 ± 1.17	5.7 (5.2–6.7)	** < 0.001**^a*^
*P (mg/dl)*	3.79 ± 2.45	4 (1.5–6)	2.19 ± 2.08	1.7 (0.1–3.8)	**0.002**^a*^
*TOS* *(μmol H*_*2*_*O*_*2*_*/L)*	3 ± 1.88	2.4 (1.7–3.6)	1.92 ± 1.52	1.3 (1–2.6)	** < 0.001**^a*^
*TAOC* *(mmol Trolox/L)*	1.31 ± 0.92	1 (0.5–2.2)	1.79 ± 0.91	2.1 (0.7–2.6)	**0.026**^a*^
*SAA (U/ml)*	64.04 ± 13.28	65.3 (54.2–72.2)	39.38 ± 9.54	38.1 (32.2–46.2)	** < 0.001**^b*^

*DMFT*, decayed missing filled teeth; *dft*, decayed filled teeth; *DI*, debris index; *CI*, calculus Index; *OHI-S*, simplified oral hygiene index; *UWS*, unstimulated whole salivary (mL/min); *SWS*, stimulated whole salivary flow rate (mL/min); *SBC*, salivary buffer capacity; *SpH*, salivary pH; *Cr*, creatinine; *Ca*, calcium; *K*, potassium; *P*, phosphorus; *TOS*, total oxidant status; *TAOC*, total antioxidant capacity; *SAA*, salivary α-amylase. ^a^Mann–Whitney *U* test. ^b^Welch’s *T*-test. ^c^Chi-square test. Two-tailed *p*-value *significant at 0.05 level

**Fig. 1:**
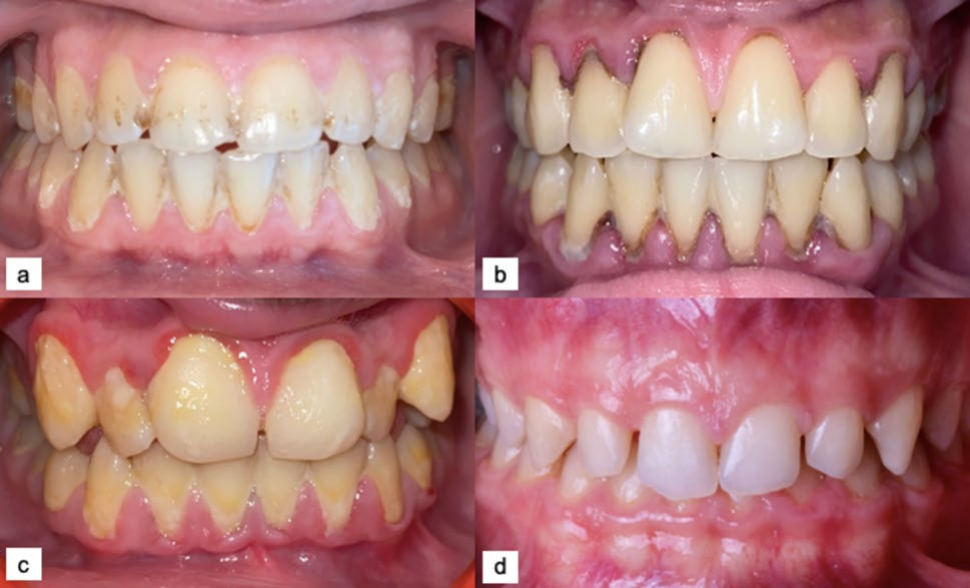
High OHI-S score due to extensive debris and calculus accumulation in 14- (**a**), 13- (**b**), and 11- (**c**) year-old stage 4–5 CKD patients. OHI-S score of zero due to the absence of debris and calculus accumulation in a 10-year-old healthy child (**d**) (OHI-S, simplified oral hygiene index)

Oral health status, salivary, and plasma characteristics in different stages of CKD are compared in Table 3. Patients in stages 1–3 (*n* = 14, 32.6%) and stages 4–5 (*n* = 29, 67.4%) had similar scores for DMFT, DI, CI, and OHI-S indices (*p* > 0.05). The dft score was significantly lower in children in stages 4–5 compared to those in stages 1–3 (*p* < 0.05). Intraoral photographs of participants in the study are shown in Fig. 1. High OHI-S scores were observed in CKD patients aged 14 (a), 13 (b), and 11 (c) years due to extensive debris and calculus accumulation, whereas a low OHI-S score was observed in the 10-year-old healthy patient (d), where no debris or calculus was present. The stimulated whole salivary flow rate and plasma Cr levels were significantly higher in children in stages 4–5 compared to those in earlier stages (*p* < 0.05). The remaining salivary characteristics and plasma levels showed no significant differences between the earlier and later stages (*p* > 0.05).

**Table 3:** Oral health parameters, salivary, and plasma characteristics in children with different stages of chronic kidney disease (*n* = 43)

	Stages 1–3 (*n* = 14)		Stages 4–5 (*n* = 29)		
Variables	*Mean* ± *SD*	*Median (IQR)*	*Mean* ± *SD*	*Median (IQR)*	*p*-value
*Oral health parameters*					
*DMFT*	0.21 ± 0.8	0 (0–0)	1.14 ± 1.71	0 (0–3)	0.053^a^
*dft*	2.86 ± 3.63	0.5 (0–7)	0.59 ± 1.48	0 (0–0)	**0.015**^a*^
*DI*	1.6 ± 0.71	1.8 (1.5–2)	1.66 ± 0.57	1.7 (1.2–2.2)	0.948^a^
*CI*	0.88 ± 0.7	1 (0.2–1.5)	1.11 ± 0.69	1.3 (0.5–1.5)	0.340^a^
*OHI-S*	2.48 ± 1.3	2.9 (1.7–3.7)	2.81 ± 1.23	2.8 (1.8–4)	0.435^a^
*Salivary characteristics*					
*UWS (mL/min)*	0.26 ± 0.08	0.3 (0.2–0.3)	0.33 ± 0.18	0.3 (0.2–0.4)	0.404^a^
*SWS (mL/min)*	0.43 ± 0.11	0.4 (0.4–0.5)	0.59 ± 0.22	0.6 (0.4–0.8)	**0.024**^a*^
*SBC*	9.36 ± 2.9	10 (9–12)	10.52 ± 1.27	10 (10–12)	0.402^a^
*SpH*	7.33 ± 0.51	7.4 (7.2–7.8)	7.46 ± 0.23	7.4 (7.4–7.6)	0.751^a^
*Urea(mg/dl)*	13.75 ± 8.11	14.3 (5.1–21.6)	11.6 ± 6.99	11.8 (5–17.2)	0.434^a^
*Cr (mg/dl)*	7.87 ± 1.24	7.6 (7.3–8.5)	7.62 ± 1.98	7.4 (6.1–8.6)	0.612^c^
*Ca (mg/dl)*	4.47 ± 2.43	3.4 (3–5.7)	3.74 ± 2.15	3.1 (2.3–5)	0.259^a^
*K (mg/dl)*	9.54 ± 3.77	7.8 (7.2–8.9)	9.34 ± 3.98	7.5 (7.1–10.5)	0.641^a^
*P (mg/dl)*	3.56 ± 2.36	2.8 (1.5–6)	3.9 ± 2.53	4.4 (1.5–5)	0.887^a^
*TOS (μmol H*_*2*_*O*_*2*_*/L)*	3.16 ± 2.12	2.4 (2–3.4)	2.93 ± 1.79	2.6 (1.6–3.6)	0.756^a^
*TAOC (mmol Trolox/L)*	1.31 ± 0.97	0.9 (0.4–2.2)	1.31 ± 0.91	1.1 (0.6–2.2)	0.888^a^
*SAA (U/ml)*	62.96 ± 10.19	65.1 (54.7–68.1)	64.57 ± 14.67	65.3 (54.2–74.3)	0.714^*b*^
*Plasma*					
*Urea (mg/dl)*	36.79 ± 26.69	32 (16–45)	49.17 ± 23.1	50 (36–59)	0.052^a^
*CR (mg/dl)*	7.25 ± 15.15	1.4 (1.0–2.0)	4.46 ± 2.25	4.8 (2.5–5.7)	**0.003**^a*^
*Ca (mg/dl)*	16.06 ± 23.88	9.6 (9.4–10.3)	9.67 ± 0.64	9.5 (9.2–10.1)	0.621^a^
*P (mg/dl)*	5.16 ± 0.91	4.9 (4.5–5.7)	5.52 ± 1.41	5.3 (4.4–6.5)	0.399^b^

*DMFT*, decayed missing filled teeth; *dft*, decayed filled teeth; *DI*, debris index; *CI*, calculus Index; *OHI-S*, simplified oral hygiene index; *UWS*, unstimulated whole salivary (mL/min); *SWS*, stimulated whole salivary flow rate (mL/min); *SBC*, salivary buffer capacity; *SpH*, salivary pH; *Cr*, creatinine; *Ca*, calcium; *K*, potassium; *P*, phosphorus; *TOS*, total oxidant status; *TAOC*, total antioxidant capacity; *SAA*, salivary α-amylase. ^a^Mann–Whitney *U* test. ^b^Independent samples *T*-test. ^c^Welch’s *T*-test. Two-tailed *p*-value *significant at 0.05 level

The salivary urea in children with CKD was negatively correlated with plasma urea (r_s =  − 0.361; *p* = 0.017) and plasma Cr (r_s =  − 0.358; *p* = 0.019) levels. The salivary urea level in children with CKD tends to decrease as plasma urea and Cr levels increase. The salivary Cr, Ca, K, P, TOS, TAC, and SAA levels were not significantly correlated with plasma levels or oral health parameters (Table 4). The later stages of CKD were associated with lower dft scores (r_s =  − 0.350; *p* = 0.022) and higher levels of plasma urea (r_s = 0.529; *p* < 0.001) and plasma Cr (r_s = 0.615; *p* < 0.001) (Table 4).

**Table 4:** Correlation between salivary biomarker levels and plasma biomarker levels and oral health parameters in children with chronic kidney disease (*n* = 43)

	*Saliva*									
Variables		*Urea (mg/dl)*	*Creatinine (mg/dl)*	*Calcium (mg/dl)*	*Potassium (mg/dl)*	*Phosphorus (mg/dl)*	*TOS (μmol H*_*2*_*O*_*2*_*/L)*	*TAOC (mmol Trolox/L)*	*SAA (U/ml)*	*CKD level*
*Plasma*										
*Urea (mg/dl)*	***rho***	− 0.361*	0.077	− 0.096	0.206	0.292	− 0.007	− 0.141	0.034	0.529*
	**p**	**0.017**	0.625	0.539	0.185	0.057	0.962	0.369	0.826	** < 0.001**
*Creatinine (mg/dl)*	***rho***	− 0.358*	− 0.056	− 0.009	0.115	0.168	− 0.034	− 0.130	− 0.086	0.615*
	**p**	**0.019**	0.723	0.956	0.464	0.281	0.830	0.404	0.583	** < 0.001**
*Calcium (mg/dl)*	***rho***	0.070	0.005	0.123	− 0.165	− 0.174	0.031	− 0.131	− 0.114	0.091
	**p**	0.656	0.973	0.434	0.291	0.264	0.846	0.404	0.466	0.560
*Phosphorus (mg/dl)*	***rho***	− 0.229	0.094	0.219	− 0.077	− 0.006	0.212	− 0.080	0.043	0.244
	**p**	0.139	0.550	0.158	0.621	0.967	0.172	0.609	0.787	0.115
*DMFT*	***rho***	0.158	0.017	− 0.039	− 0.062	0.127	0.252	0.076	0.249	0.282
	**p**	0.312	0.915	0.803	0.693	0.418	0.103	0.627	0.107	0.067
*dft*	***rho***	0.136	0.117	− 0.009	0.148	− 0.172	0.147	− 0.080	0.145	− 0.350*
	**p**	0.386	0.456	0.955	0.345	0.269	0.348	0.612	0.352	**0.022**
*DI*	***rho***	− 0.086	− 0.006	− 0.019	− 0.105	0.007	− 0.023	0.056	0.146	0.030
	**p**	0.583	0.971	0.905	0.505	0.966	0.885	0.723	0.349	0.850
*CI*	***rho***	0.002	0.097	0.011	− 0.226	− 0.047	− 0.033	0.249	0.166	0.141
	**p**	0.989	0.534	0.943	0.145	0.766	0.833	0.108	0.287	0.367
*OHI-S*	***rho***	− 0.062	− 0.004	− 0.012	− 0.159	− 0.013	− 0.033	0.149	0.122	0.120
	**p**	0.691	0.979	0.939	0.307	0.933	0.834	0.341	0.438	0.444

*DMFT*, decayed missing filled teeth; dft, decayed filled teeth; *DI*, debris index; *CI*, calculus Index; *OHI-S*, simplified oral hygiene index; *TOS*, total oxidant status; *TAOC*, total antioxidant capacity; *SAA*, salivary α-amylase. Spearman’s rho correlation coefficient two-tailed *p*-value *significant at 0.05 level

## Discussion

The present study is the first to comprehensively investigate the salivary and intraoral findings associated with CKD in pediatric patients at different stages. In this study, we found that Ca and TAOC levels in the unstimulated saliva of children with CKD were lower, while pH, urea, Cr, K, P, TOS, and SAA levels were higher compared to healthy children. DMFT/dft scores were lower in children with CKD compared to healthy children, whereas DI, CI, and OHI-S scores were higher, and these differences were statistically significant. Consequently, the primary null hypothesis of the study was rejected. The secondary null hypothesis was partially rejected due to significant correlations observed between salivary urea and plasma urea and Cr levels.

In line with previous studies [18, 25, 26], we observed substandard oral conditions and inadequate oral hygiene among children with CKD, consistent with the findings of Andaloro et al. [27], who reported that children with CKD exhibit a lower prevalence of oral hygiene practices compared to their healthy peers. Conversely, Pham et al. [18] found no significant difference in oral hygiene habits between individuals with CKD and healthy controls. These variations in oral hygiene habits are often attributed to the challenges of managing CKD and its comorbidities, which may limit the time and resources available for oral care [3]. As CKD progresses, the therapeutic burden increases, often resulting in the neglect of oral hygiene care. Sezer et al. [9] and Davidovich et al. [28] noted elevated mean DI, CI, and OHI-S scores in children at later stages of CKD. In our study, while significant differences in these scores were found between children with CKD and their healthy counterparts, no notable variation was observed across different CKD stages. This could be attributed to the individualized treatment regimens and variations in oral hygiene practices influenced by the children’s overall health status.

Serni et al. [26] reported that the increase in OHI-S values among individuals with CKD can be attributed not only to calcium–phosphorus imbalances, changes in salivary pH, flow rate, buffering capacity, and biochemical composition, but also to decreased attention to oral hygiene due to the patients’ overall health condition. Similarly, Rodrigues et al. [13] and Lasisi et al. [29] identified a strong correlation between salivary parameters and the likelihood of developing caries and periodontal disease in individuals with CKD. In contrast, while our study detected significant alterations in the salivary composition of children with CKD, no significant relationship was found between these changes and dental caries or oral hygiene scores. This finding challenges the conventional understanding that reduced salivary flow and inadequate oral hygiene increase the risk of caries, suggesting that the altered salivary composition in CKD patients may play a role in mitigating this risk.

In recent years, significant advancements in pediatric nephrology have increased the life expectancy of children with kidney disease [3]. Findings from the limited studies investigating the salivary and oral manifestations of the disease indicate that CKD increases susceptibility not only to cardiovascular diseases but also to olfactory and taste disorders, periodontal diseases, halitosis, and salivary gland dysfunction [3, 30–32]. Hyposalivation is among the most common oral manifestations of CKD and is a contributing factor to progressive dental caries [3]. Considering the reduced salivary flow, poor oral hygiene, and cariogenic dietary habits, a higher prevalence of caries might be expected in these children compared to healthy peers [27]. However, the high urea levels in the saliva of individuals with CKD, which neutralize plaque and exhibit antibacterial properties, lead to increased ammonia concentration, improved pH, and enhanced buffering capacity [33]. Pakpour et al. [34] reported a significantly higher incidence of caries in hemodialysis patients, whereas a longitudinal study by Nylund et al. [35], which followed patients from predialysis to kidney transplantation, found no difference in DMFT scores between the groups. Conversely, Limeira et al. [36] suggested that saliva in individuals with CKD may possess protective properties against dental caries. Similarly, Silva et al. [37] observed a lower incidence of dental caries in children with CKD compared to their healthy counterparts, a finding consistent with the results of our study. In our study, elevated salivary urea levels were observed to contribute to a lower prevalence of dental caries by increasing salivary pH and buffering capacity.

Studies have reported decreased salivary flow rates and alterations in salivary components in patients with CKD, including those undergoing hemodialysis for renal failure [3, 38]. Children with CKD exhibited significantly reduced salivary flow rates compared to healthy controls, consistent with the findings of Maciejczyk et al. [15], who emphasized that salivary gland dysfunction associated with CKD adversely affects both saliva production and composition. Unstimulated salivary gland function can be evaluated through measurements of salivary flow rate and SAA activity. SAA, produced by the salivary glands, is the primary protein in saliva, accounting for up to 50% of total salivary protein [38]. In our study, statistically significant differences were observed in these parameters between children with CKD and healthy controls, suggesting that kidney disease may impair salivary gland function. Szulimowska et al. [14] found higher salivary concentrations of SAA in children with CKD compared to healthy controls. Consistent with our findings, Tomas et al. [39] and Romero et al. [40] reported increased concentrations of serum, pancreatic, and salivary alpha-amylase in patients at different stages of CKD and in those undergoing hemodialysis. Tomas et al. [39] proposed that these elevations might be associated with the severity of renal failure and/or hemodialysis treatment. However, the relationship between these parameters and oral health status remains unclear.

The buffering capacity of saliva, composed of three main systems—carbonic acid/bicarbonate, phosphorus, and protein—is a critical factor in maintaining the pH balance of the oral environment [41]. Elhusseiny et al. [6] reported that despite reduced salivary flow, patients with CKD exhibited increased salivary pH and buffering capacity, attributed to elevated levels of urea and phosphorus diffused passively from serum into saliva. Similarly, Velan et al. [3] observed increased salivary pH and buffering capacity in CKD patients despite reduced salivary flow. Consistent with these findings, our study also suggests that the elevated buffering capacity and pH levels in children with CKD may reflect mechanisms that provide resistance against acidogenic changes in the oral environment.

Oxidative stress occurs in uremic conditions associated with renal failure when the balance between antioxidants and oxidants shifts in favor of oxidants [42]. Imbalances in the salivary oxidative and antioxidant systems may contribute to oral diseases, including periodontal diseases and dental caries [43]. Antioxidants present in human saliva play a crucial role in mitigating oxidative stress by neutralizing free radicals and reactive oxygen species [16]. Previous studies have shown that oxidative stress parameters are elevated, while antioxidant defense systems are diminished in the serum of individuals with CKD [15, 44]. Consistent with these findings, our research revealed higher salivary TOS levels and lower TAOC levels in children with CKD compared to healthy children. This suggests that CKD negatively affects antioxidant defense mechanisms, leading to increased oxidative stress and insufficient antioxidant capacity. The reduced TAOC levels indicate a heightened potential for cellular damage in these patients [45].

In patients with CKD, a positive correlation has been observed between urea and Cr levels measured in blood serum and saliva [46, 47]. However, in our study, a negative correlation was found between salivary urea and Cr levels and plasma urea and Cr levels, and this relationship was statistically significant. This discrepancy may be attributed to the time difference between the collection and measurement of saliva and its comparison with blood serum. Salivary biomarkers are subject to dynamic fluctuations, and saliva analysis requires specialized equipment, making it less routine and accessible compared to blood tests. In conclusion, the negative and significant correlation between salivary urea and Cr levels and plasma urea and Cr levels is a notable finding. It underscores the potential of saliva as a non-invasive diagnostic tool and offers valuable insights into the dynamics of urea and Cr in body fluids [48].

In our study investigating the impact of kidney disease on specific serum parameters, a positive correlation was observed between plasma urea and Cr levels and CKD disease stage. This finding aligns with the results of Lin et al. [49], who reported a gradual increase in plasma urea and Cr levels with increasing disease severity. The absence of a similar positive correlation between plasma and salivary levels of these biomarkers in our study suggests that while plasma markers directly reflect disease progression, salivary biomarkers may provide a more complex representation of the disease. This complexity may be influenced by factors such as individual salivary gland function and the metabolic processes associated with CKD. Salivary urea, Cr, Ca, K, and P are important biomarkers of oral and dental health in CKD [13]. In the current study, consistent with the findings of Poposki et al. [8] and SaiKiran et al. [50], salivary urea, Cr, K, and P levels were higher in children with CKD, while salivary Ca levels were lower compared to healthy children. Although no association was found between salivary components and oral health parameters in our study, it is important to consider that the salivary biomarkers examined are not exclusive to kidney disease. The diagnostic value of salivary parameters may be limited, particularly in pediatric patients, due to salivary gland dysfunction or other disease-related medical factors. Further research involving a larger pediatric population is needed to explore disease progression, changes in salivary parameters, and their potential impact on oral health.

This study has several limitations that should be acknowledged. The lack of blinding for the single dental examiner and the absence of intra-rater reliability assessment may have introduced variability in the evaluations. Poorer oral hygiene routines and infrequent dental visits in the CKD group could have contributed to higher plaque and calculus scores, independent of the disease process. Additionally, participants were not matched for socio-economic status, a recognized determinant of oral health, which might have influenced the outcomes. The absence of preliminary studies on this topic limited the opportunity to refine the study design and methodology. Furthermore, the non-use of orthopantomograms and bitewing radiographs, which are more effective for detecting interproximal caries, represents a methodological shortcoming. Lastly, the duration of CKD was not evaluated, despite its potential impact on oral health outcomes such as caries prevalence. These limitations should be considered in the interpretation of the findings and addressed in future research to enhance understanding of the interplay between CKD and oral health.

## Conclusions

In conclusion, children with CKD exhibit lower dental caries prevalence but worse oral hygiene scores compared to their healthy peers. Additionally, they show distinct salivary biomarker levels compared to healthy children. Many of these oral health issues can be addressed through early diagnosis, palliative measures, and appropriate dental interventions. Thus, it is crucial to emphasize the importance of collaboration between dental and medical professionals in assessing and managing oral health during the treatment of CKD. We believe that further comprehensive studies with larger sample sizes are needed to raise awareness and improve understanding of this issue.

## Supplementary Information

Below is the link to the electronic supplementary material.

Graphical Abstract (PPTX 78 KB)

## Data Availability

The data that support the findings of this study are available from the corresponding author (N.K.D.) upon reasonable requests.
