# Supplementary material for: Dental caries, oral hygiene and salivary characteristics in children with chronic kidney disease: a case–control study
Source: Pediatr Nephrol. 2025 Mar 19;40(8):2627–37. doi: 10.1007/s00467-025-06730-4 (PMC12187895; doi:10.1007/s00467-025-06730-4)
Supplement: Supplementary file 1 — Graphical Abstract (PPTX 78 KB) [file 467_2025_6730_MOESM1_ESM.pptx]

## Slide 1
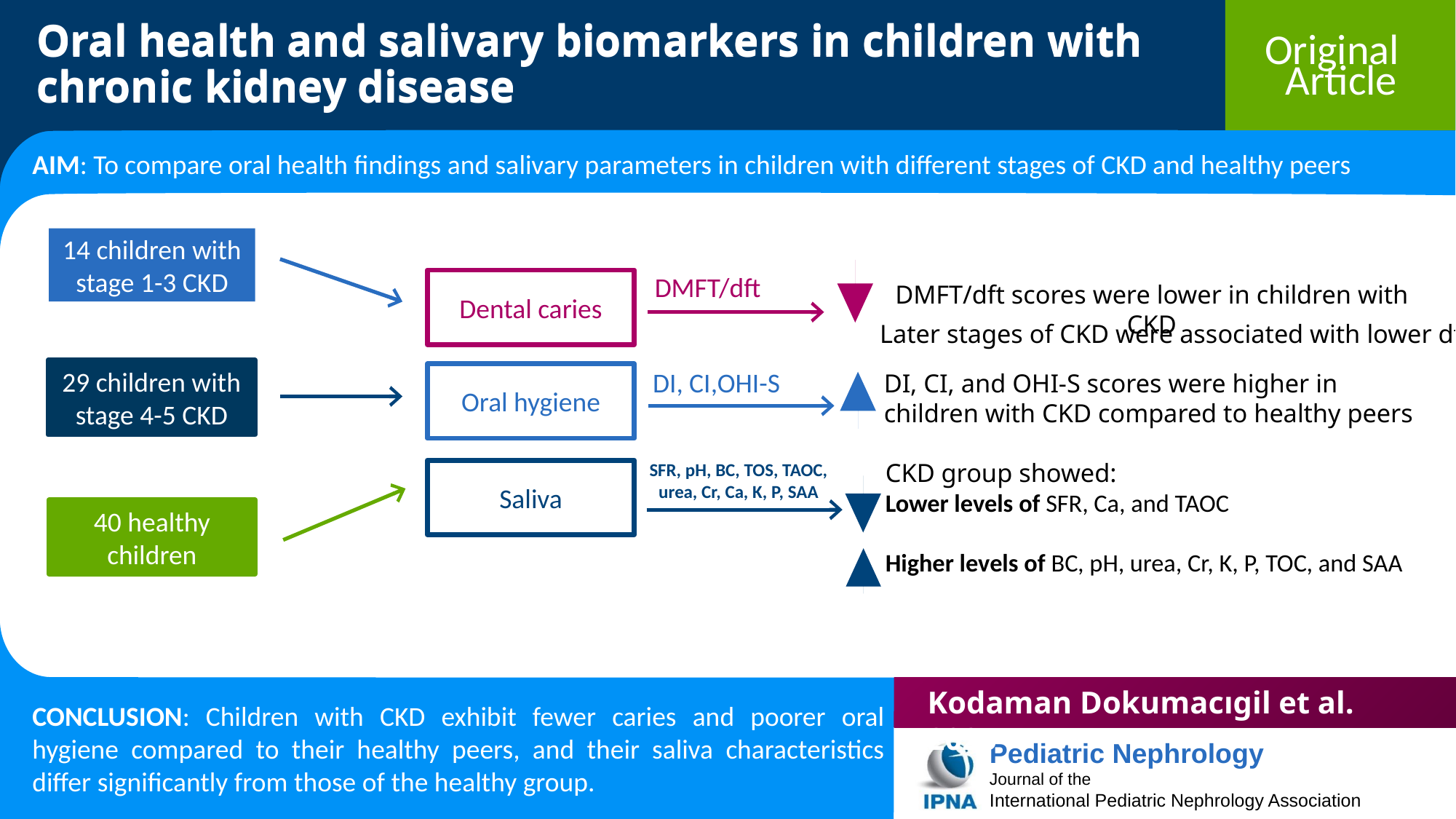

Oral health and salivary biomarkers in children with chronic kidney disease
Oral health and salivary biomarkers in children with chronic kidney disease
AIM: To compare oral health findings and salivary parameters in children with different stages of CKD and healthy peers
14 children with stage 1-3 CKD
DMFT/dft
Dental caries
DMFT/dft scores were lower in children with CKD
Later stages of CKD were associated with lower dft
29 children with stage 4-5 CKD
DI, CI,OHI-S
DI, CI, and OHI-S scores were higher in children with CKD compared to healthy peers
Oral hygiene
CKD group showed:
Lower levels of SFR, Ca, and TAOC
Higher levels of BC, pH, urea, Cr, K, P, TOC, and SAA
SFR, pH, BC, TOS, TAOC, urea, Cr, Ca, K, P, SAA
Saliva
40 healthy children
Kodaman Dokumacıgil et al. 2025
CONCLUSION: Children with CKD exhibit fewer caries and poorer oral hygiene compared to their healthy peers, and their saliva characteristics differ significantly from those of the healthy group.
